# Comparative analysis of metals, pesticides, mycotoxins, microbial contaminants and THC potency in illegal and regulated cannabis inflorescences in Canada

**DOI:** 10.1186/s42238-026-00414-y

**Published:** 2026-02-23

**Authors:** Quinton Fiering, Jordyn Mackey, Hayline Kim, Yousef Risha, Ryan Denkert, Monica Iuogovaz, Gabriela Zorila, Julia Killorn, Gayel Abi-Yaghi, Emmanuelle Bahl, Josée Verreault, Marick Gagnon, Carl Rodrigue, Roxane Arsenault, Julio Bran Barrera, Maude Lord, Jamile Ahmarani, Diana Becerra Rodriguez, Arianne Gauthier, Diana Bran Barrera, Cathy St-Laurent, Guillaume Massicotte, Roland Yang, Morgan Popplewell, Michel Blais, Peter Bradley, Cecile Le, William Mohan, André Robichaud, Catherine Deschenes, Isabelle Disnard, Geneviève Clément, Maryse Fontaine, Irène Iugovaz, Julie Bellemare, David R. Blais

**Affiliations:** 1 Pesticide Laboratory, Regulatory Operations and Enforcement Branch, Health Canada, Ottawa, Ontario K1A 0C6 Canada; 2 Microbiology Laboratory, Regulatory Operations and Enforcement Branch, Health Canada, Longueuil, Québec J4K 1C7 Canada; 3 Cannabis Laboratory, Regulatory Operations and Enforcement Branch, Health Canada, Longueuil, Québec J4K 1C7 Canada

**Keywords:** Cannabis, Flowers, Legal, Illegal, THC, Arsenic, Cadmium, Lead, Mercury, Mold, Pesticides

## Abstract

**Background:**

Following the legalization of recreational cannabis in Canada under the Cannabis Act (2018), regulatory frameworks were implemented to ensure product safety, quality, and consistency within the legal market. Previous studies revealed significantly greater pesticide contamination in illegal cannabis than in legal products. In light of these findings, the present study expands contaminant surveillance to include the quantification of Δ⁹-tetrahydrocannabinol (THC) and a broader range of potential toxicants, including metals, pesticides, mycotoxins, and microbial agents.

**Methods:**

Fifty legal cannabis products were purchased across Canadian provinces, and 50 illegal samples were obtained via law enforcement seizures. All the samples were tested via validated, accredited/attested methods. THC content was assessed using LC-UV-MS. Pesticides were analyzed via LC-MS/MS and GC-MS/MS; metals via ICP-MS/MS; mycotoxins via LC-MS/MS; and microbial contamination using MALDI-TOF.

**Results:**

THC levels in 48% of legal products deviated by more than 20% from their labeled concentrations. Microbiological testing revealed that 20% of legal products exceeded the European Pharmacopoeia microbial limits, prompting appropriate compliance and enforcement measures to address each of these cases. In contrast, 55% of the illegal products exceeded the aerobic plate count thresholds, and 73% surpassed the yeast and mold limits. Mycotoxins were undetected in legal products but were present in 12% of illegal samples. Pesticide residues were found at trace levels (0.01 µg/g) for myclobutanil and dichlobenil in two legal samples, whereas 94% of illegal samples contained pesticides, averaging 3.4 compounds per sample across 24 unique active ingredients. Metal analysis revealed higher levels of arsenic, cadmium, lead, and mercury in illegal products than in their legal counterparts. However, legal samples presented higher chromium concentrations, with peak values approximately threefold greater than those observed in illegal cannabis. The concentrations of arsenic, barium, chromium, copper, molybdenum, nickel, and vanadium exceeded the inhalation concentration limits found in Table 2 of USP <232> of the United States Pharmacopeia (USP) in one or both product categories.

**Conclusion:**

These findings demonstrate that legal THC levels differ from their label claims as well as that contaminants differ between legal and illegal cannabis products. The results provide evidence to inform regulatory oversight, enhance risk assessment efforts, and support informed decision-making by consumers and policymakers in the context of a legal cannabis framework.

**Supplementary Information:**

The online version contains supplementary material available at 10.1186/s42238-026-00414-y.

## Background

In 2018, Canada legalized the recreational use of cannabis, supplementing the existing medical cannabis framework that had been in place since 2001. The enactment of the Cannabis Act (Cannabis Act, 2018) and its associated regulations (Cannabis Regulations, 2018) sought to standardize and enforce consistent health and safety standards throughout Canada’s legal cannabis industry. To protect public health, Health Canada regulates microbial and chemical contaminants in cannabis products. Under the Cannabis Regulations, mandatory analytical testing requirements obligate license holders to demonstrate that contaminant levels do not exceed established thresholds and that the cannabis product label indicates an accurate quantity of THC and CBD.

Building on our recent findings that revealed a high prevalence and elevated levels of pesticides in illegal cannabis inflorescence compared with legal market samples (Gagnon et al. 2023), this study aims to comprehensively quantify both THC levels and a broader range of contaminants. These include metals, mycotoxins, and microbial contaminants, thereby extending the scope of analysis beyond pesticides alone.

## Methods^1^

### Sampling

To reflect as realistically as possible the sources of cannabis inflorescence available to Canadians across the country, 50 legal products (with packaging dates ranging from October 2022 to September 2023) were purchased in 2023 from 50 license holders located in five Canadian regions (British Columbia, Prairies, Ontario, Quebec, and Atlantic) (Table 1), targeting THC products (with little/no CBD and excluding prerolled products) containing up to approximately 250 mg/g total THC. The 50 illegal cannabis samples were obtained from seizures by law enforcement officers across the country and submitted to Health Canada for laboratory testing in 2023.

**Table 1:** Geographical distribution of cannabis (*C. sativa*) inflorescence samples obtained across Canada

Region	Legal samples	Illegal samples
British Columbia	11	23
Prairies	8	2
Ontario	10	12
Québec	12	8
Atlantic	9	5
Total	50	50

### Reagents and consumables

Analytical grade acetone and toluene were purchased from EMD Millipore (Darmstadt, Germany). Analytical grade acetonitrile, methanol and Na_2_SO_4_ were purchased from Fisher Scientific (Fairlawn, NJ). Water was obtained from a Milli-Q^®^ Plus Ultra Pure Water system (Millipore Corp., Burlington, MA). Strata™C18-E was obtained from Phenomenex (Torrance, CA). Supelclean™ ENVI™-Carb SPE Tubes were obtained from Supelco (Bellefonte, PA). Sep-Pak^®^ Classic NH2 Cartridges were obtained from Waters Corp. (Milford, MA).

### Total THC analysis

Cannabis inflorescence samples were homogenized via a laboratory-grade homogenizer (2010 Geno/grinder, SPEX SamplePrep, Metuchen, NJ, USA). The samples (0.3 g) were subjected to extraction in 30 mL of methanol with continuous agitation for 30 min, sonicated for 15 min, and centrifuged at 3000 RPM for 5 min. The supernatant was diluted appropriately to ensure that the analyte concentrations fell within the calibrated dynamic range. (±)-11-Nor-9-carboxy-Δ9-tetrahydrocannabinol-D_3_ was spiked into each extract prior to analysis as an internal standard. Quantitative analysis was performed via an ACQUITY QDa UPLC H-Class system couple with a single quadrupole mass spectrometer (Waters Corp., Milford, MA, USA). Chromatographic separation was achieved on a Acquity HSS T3 1.8 μm, 2.1 × 150 mm C18 reversed-phase column (Waters Corp., Ireland) employing water‒acetonitrile gradient elution. Cannabinoid identification was confirmed by matching retention times, UV absorption spectra, and accurate mass measurements. Quantification was possible with signals from both the UV and MS detectors. The total THC content represents the sum of Δ9-tetrahydrocannabinol (Δ9-THC) and its acidic precursor Δ9-tetrahydrocannabinolic acid (Δ9-THCA), accounting for the potential decarboxylation of Δ9-THCA to Δ9-THC during sample processing. The method LOD and LOQ were 0.25 mg/g and 2.5 mg/g respectively, for both d9-THC and d9-THCA.

### Metal analyses

Cannabis inflorescence samples, without prior drying, were cryogenically homogenized via a freezer mill (Spex sample prep 6875D, SPEX, New Jersey, USA) prior to subsampling. Encapsulated steel impactor with polycarbonate and polycarbonate end plug were use to prevent any metal contamination from stainless steel. The samples were subjected to closed-vessel microwave-assisted (Mars 6 System, CEM, North Carolina, USA) acid digestion via a mixture of nitric acid (HNO₃) : hydrochloric acid (HCl) (7:1) (ecoTRACE^PPT^ ultra-pure grade with certified impurities less than 10 ppt, package in FEP polymer bottles, GFS Chemicals, Columbus, OH, USA) at 210 °C in 55 mL Teflon (TFM and or PFA) vessels. The extracts were then diluted to appropriate volumes, and internal standards were introduced via in-line addition. All Polypropylene and Polystyrene laboratory glassware used while working with the sample went to a multistep rigorous rinse and overnight soaking in acids solutions process prior to use. Eighteen metals were selected for compliance monitoring by Health Canada based on other jurisdictions where recreational cannabis has been legalized and based on toxicological risk and likelihood of occurrence as per the United States Pharmacopeia 232 (Zaidi 2010). Analysis targeting the 18 metals was validated with calibration curves spanning from one-tenth to twice the specified limit concentrations of the United States Pharmacopeia 232 (Zaidi 2010). Detection and quantification were carried out via an Inductively Coupled Plasma Triple Quadrupole mass spectrometer (ICP-QQQ, Agilent Technologies, Santa Clara, CA), with various collision/reaction cell gas modes to increase the selectivity and sensitivity for specific elements. Analysis of Li in single quadrupole (MS) was perform in No Gas mode, analysis of Co, Cr, Ni, Cu, Mo, Ag, Sn, Sb, Ba, Au, Hg, Tl and Pb also in single quadrupole (MS) were perform in collision mode (Helium), in reaction mode Se was analyzed with H_2_ gas in (MS/MS) and Cd, As, V with (H_2_ + O_2_) gas in (MS/MS). All the modes in Standard Resolution and dwell time was adjusted according to the concentration levels. The precise order of analysis modes was optimized as follow : No Gas/H_2_ + O_2_/H_2_/He. The method LOQs are listed in Supplementary Tables 1 and 2.

### Pesticide analyses

Pesticide analyses were analyzed identically as previously described in (Gagnon et al. 2023). In summary, cannabis inflorescence samples were homogenized and extracted with acetonitrile, followed by mechanical shaking, centrifugation, and multi-step cleanup using dispersive C18 and SPE cartridges. Extracts were concentrated, solvent-exchanged, and spiked with internal standards. Samples were split for parallel GC–MS/MS and LC–MS/MS analysis. LC–MS/MS used a C18 column with gradient elution and positive electrospray ionization, while GC–MS/MS employed splitless injection, temperature-programmed separation, and electron impact ionization. Both instrument methods used multiple reaction monitoring to obtain qualitative and quantitative analyte data under optimized instrumental conditions. Each sample was analyzed in triplicate with the default 50% expanded uncertainty value for multi-residue methods. Method validation and limits of detection (LOD) are provided in (Gagnon et al. 2023).

### Mycotoxins

Analyses of aflatoxins B1, B2, G1 and G2, ochratoxin A and deoxynivalenol were carried as per the methods described by (Desaulniers Brousseau et al. 2024). In summary, cannabis inflorescence was ground, and ~ 1 g weighed into a 50 mL tube and spiked with analytical standards. The sample was extracted with 10 mL of formic acid/acetonitrile/water buffer, shaken, and centrifuged. One milliliter of supernatant was cleaned by C18 solid-phase extraction (SampliQ C18 Endcapped 500 mg, Agilent Technologies, Santa Clara, CA Technologies), then diluted with mobile phases. Samples are analyzed by liquid chromatography coupled with tandem mass spectrometry (LC‒MS/MS, QTRAP 6500+, AB SCIEX, Framingham, MA, USA). Chromatography uses a C18 column (Raptor ARC-18 column − 100 mm x 2.1 mm, 2.7 μm, Restek, Bellefonte, USA) and methanol–water mobile phases with 2 mM ammonium formate and 1mL L^− 1^ formic acid as modifiers (LC-MS grade, Sigma, Burlington, MA, USA). Detection is performed by ESI-MS in positive mode, monitoring two transitions per analyte. Each sample was initially screened for the presence of mycotoxins. Samples that tested positive were reprepared and quantified using a six-point standard addition calibration curve. The method LODs were AFB1 = 0.3 ng g^− 1^, AFB2 = 0.5 ng g^− 1^, AFG1 = 0.5 ng g^− 1^, AFG2 = 0.6 ng g^− 1^, OTA = 2.2 ng g^− 1^, DON = 12 ng g^− 1^.

### Microbial contaminants

Cannabis inflorescence samples were prepared and tested for total aerobic microbial count (TAMC), total yeast and mold count (TYMC), bile-tolerant gram-negative bacteria (BTGN), and the presence or absence of *Escherichia coli* and *Salmonella spp*. according to the (European Pharmacopoeia, 2024). In addition, for the samples whose TYMC counts were greater than the limits set by (European Pharmacopoeia, 2024), the presence or absence of *Aspergillus spp*. was tested via a combination of microscopic observation and a matrix-assisted laser desorption/ionization time-of-flight (MALDI-TOF) spectrometer (Microflex LT/SH, Bruker Daltonics GmbH & Co. KG, Bremen, Germany).

### Multivariate correlation analysis

#### THC – contaminants correlation

Non-detects were treated as left-censored observations at analyte-specific limits of detection. Correlation analyses were conducted using a two-part framework, separating analyte co-occurrence (detected vs. non-detected) from concentration correlations conditional on detection. Rank-based correlation coefficients were used to account for non-normality. Analyses were stratified by legal status, and false discovery rate was controlled using the Benjamini–Hochberg procedure.

#### Contaminant correlation

Non-detects were treated as left-censored observations. Primary analyses used a two-part modeling framework separating detection probability from concentration magnitude. Detection-level associations were assessed using tetrachoric correlations, while concentration-level associations were evaluated using Spearman rank correlations on log-transformed detected values. Integrated associations were estimated using two-part Kendall’s τ. All correlation analyses were performed separately for legal and illegal samples, with false-discovery-rate correction applied to multiple comparisons. Substitution-based approaches (LOD/√2 and LOD/2) were evaluated only as sensitivity analyses.

## Results

This study expands upon previous findings (Gagnon et al. 2023) by encompassing not only pesticide residues but also metals, mycotoxins, microbial contaminants and THC levels in real-world cannabis inflorescence samples available across Canada. The objective was to determine THC potency and the prevalence of these contaminants in legal versus illegal cannabis products. A total of 100 samples, 50 legal and 50 illegal samples, were analyzed for THC content alongside metals, pesticides, mycotoxins, and microbial contaminants.

### THC levels

**Fig. 1:**
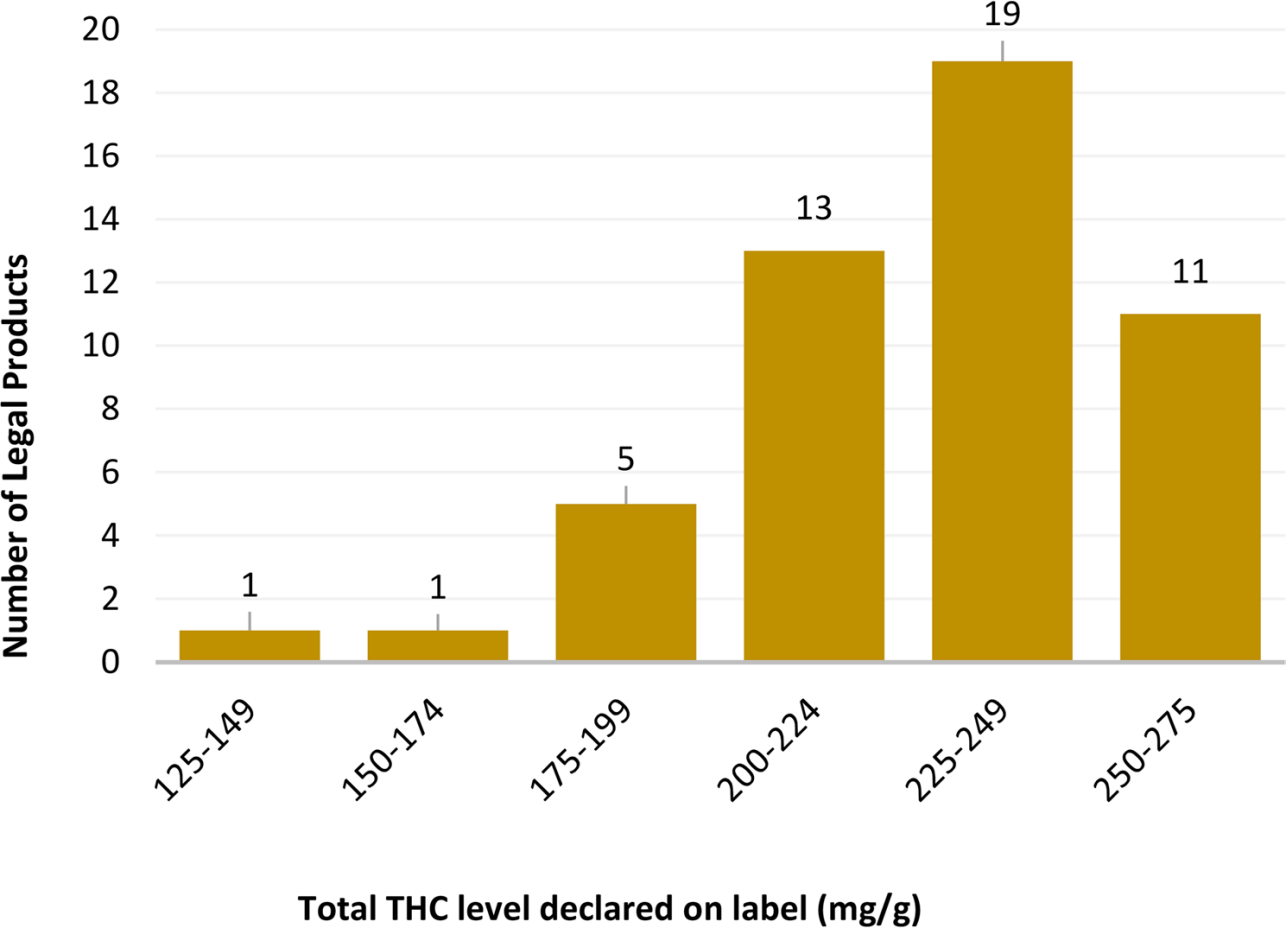
Labeled total THC levels in legal commercially available cannabis inflorescences

The total THC concentration reported on the label of legal commercially available cannabis inflorescence ranged from 143 mg/g to 261 mg/g (Fig. 1).

**Fig. 2:**
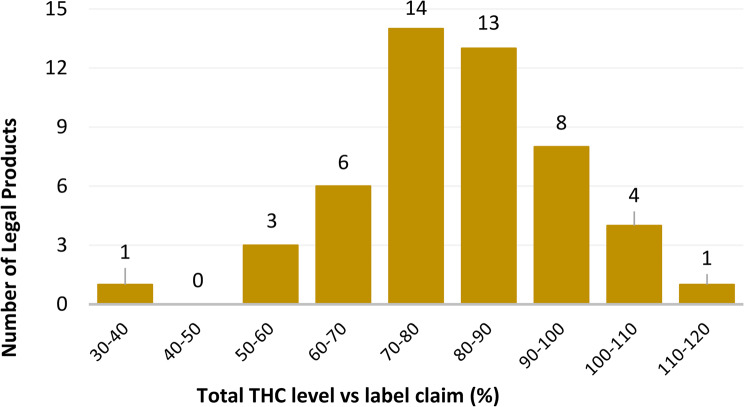
Percent of total THC levels measured versus the labeled amount in legal commercial cannabis inflorescence

A comparison of the measured total THC levels with the label claims (Fig. 2) revealed that 52% (*n* = 26) of the products contained at least 80% of their declared THC content. Conversely, 28% of the products (*n* = 14) measured between 70% and 80% of the labeled THC amount, whereas 20% (*n* = 10) contained less than 70% of the stated THC concentration.

**Fig. 3:**
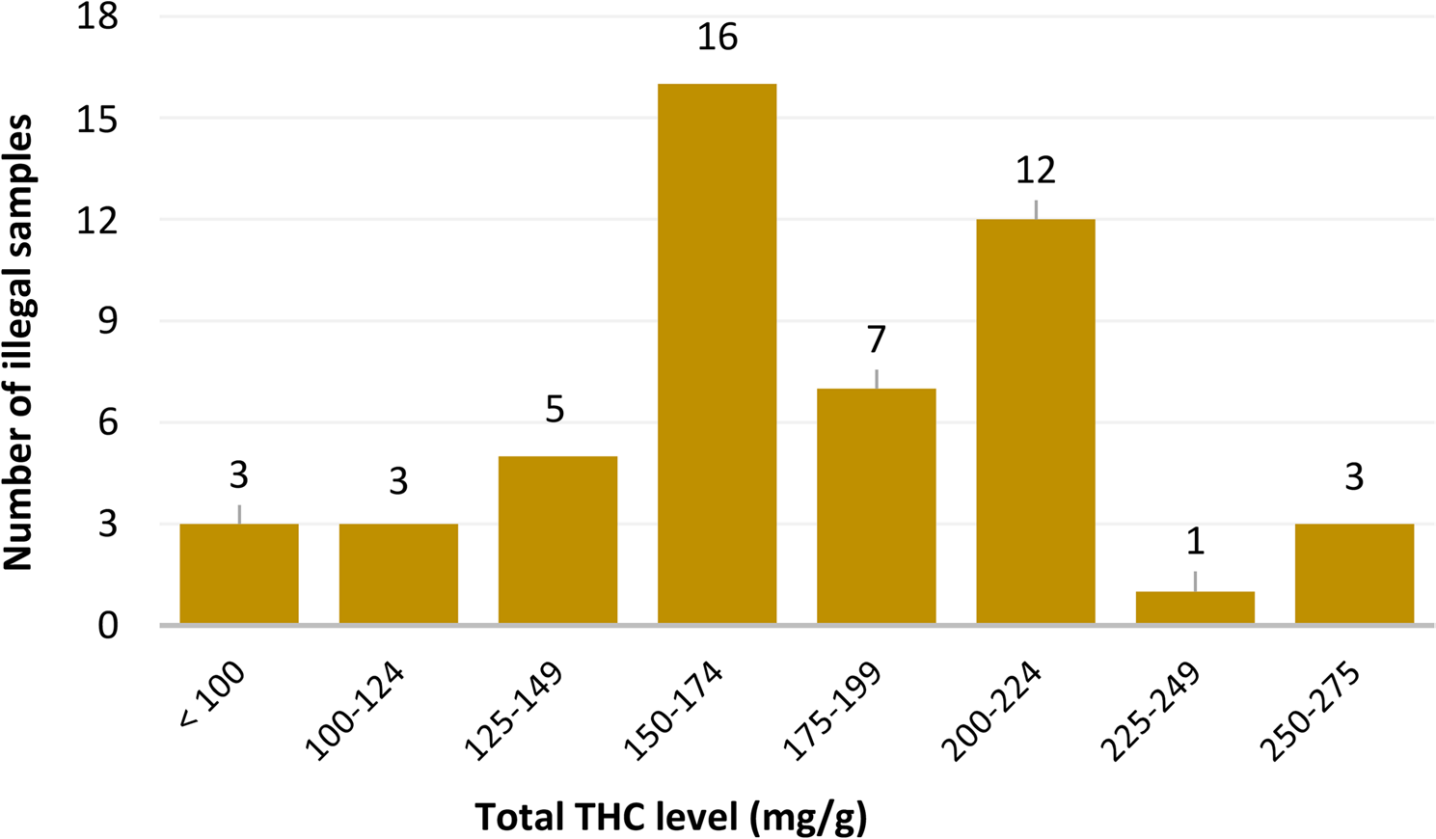
Total THC levels in illegal cannabis inflorescence

Analysis of total THC levels in illegal cannabis inflorescence revealed a broad potency range (Fig. 3). Notably, 54% of the illegal samples (*n* = 27) presented total THC concentrations below 175 mg/g. In contrast, only two legal cannabis samples presented THC levels below this threshold (Fig. 1).

### Metals

A comparison of the metals of the Class 1 metals, that are considered highly toxic under the USP <232> (Sarma et al. 2020), revealed a significantly greater prevalence of arsenic, cadmium, lead, and mercury in illegal cannabis inflorescence than in legal samples (Table 2). The maximum concentrations measured for these four metals in illegal samples were nearly double those measured in legal products (Supplementary Tables 1 and 2). Some measured concentrations of arsenic, barium, chromium, and vanadium exceeded their United States Pharmacopeia (USP) inhalation concentration limits found in Table 2 of USP <232> in one or both sample groups, whereas the majority of the copper, molybdenum, and nickel analyzed exceeded the USP concentration limits (Table 2). Antimony, gold, selenium, silver, thallium, and tin were not detected at significant levels in either illegal or legal samples, whereas copper and molybdenum were detected in nearly all the samples analyzed (Table 2, Supplementary Tables 1 and 2).

**Table 2:** Metal concentrations in legal and illegal cannabis inflorescence relative to their respective United States Pharmacopeia (USP 232) concentration limits (Sarma et al. 2020)

Metal	USP limit (µg/g) USP 232*	Legal			Illegal		
		Detection rate (%)	> USP limit (%)	Concentration range (µg/g)	Detection rate (%)	> USP limit (%)	Concentration range (µg/g)
Antimony (Sb)	2.0	0	0	< 0.20	0	0	< 0.20
Arsenic (As)	0.2	18	0	< 0.02–0.16	44	2	< 0.02–0.29
Barium (Ba)	30.0	36	2	< 3.00–40	38	2	< 3.00–95
Cadmium (Cd)	0.3	18	0	< 0.03–0.16	50	0	< 0.03–0.28
Chromium (Cr)	0.3	66	6	< 0.03–0.89	50	4	< 0.03–0.33
Cobalt (Co)	0.3	14	0	< 0.03–0.12	8	0	< 0.03–0.07
Copper (Cu)	3.0	100	100	3.7–64	100	98	2.6–24
Gold (Au)	0.3	0	0	< 0.01	0	0	< 0.01
Lead (Pb)	0.5	8	0	< 0.05–0.24	32	0	< 0.05–0.32
Lithium (Li)	2.5	8	0	< 0.25–0.76	12	0	< 0.25–1.6
Mercury (Hg)	0.1	6	0	< 0.01–0.03	12	0	< 0.01–0.06
Molybdenum (Mo)	1.0	98	74	< 0.10–9.9	100	16	0.15–2.5
Nickel (Ni)	0.6	72	2	< 0.05–4.7	78	2	< 0.05–0.91
Selenium (Se)	13.0	0	0	< 1.30	0	0	< 1.30
Silver (Ag)	0.7	0	0	< 0.07	0	0	< 0.07
Thallium (Tl)	0.8	0	0	< 0.08	0	0	< 0.08
Tin (Sn)	6.0	0	0	< 0.60	4	0	< 0.60–1.4
Vanadium (V)	0.1	22	4	< 0.01–0.36	22	6	< 0.01–0.19

* Assuming a maximum consumption of 10 g/day

### Pesticide residues

As shown in Table 3, two legal cannabis products contained trace levels of pesticides (myclobutanil and dichlobenil) measured at the lowest calibrated level (LCL) of 0.01 µg/g.

**Table 3:** Pesticide residue levels in dried cannabis

Pesticide detected	Pesticide type	Detection rate > 0.01 µg/g (%)	Concentration range (µg/g)
Legal			
Dichlobenil	Herbicide	2	0.01
Myclobutanil	Fungicide	2	0.01
Illegal			
Bifenazate	Acaricide (Miticide)	12	0.027–2.0
Boscalid	Fungicide	26	0.01–12
Chlorphenapyr	Insecticide / Acaricide	24	< 0.02–1.4
Chlorpyrifos	Insecticide	2	0.054
p, p’Dicofol	Acaricide (Miticide)	2	0.018
Dimethoate	Insecticide / Acaricide	6	0.042–0.94
Fluxapyroxad	Fungicide	2	0.018
Fluopyram	Fungicide / Nematicide	24	0.016–15
Imidacloprid	Insecticide	6	0.018–0.10
Malathion	Insecticide	2	0.022
Metalaxyl	Fungicide	4	0.042–0.14
Myclobutanil	Fungicide	72	0.01–130
n-Octyl bicycloheptene dicarboximide	Synergist	2	0.026
Oxathiapiprolin	Fungicide	2	0.018
Paclobutrazol	Plant Growth Regulator	60	0.048–2.4
Permethrin	Insecticide	10	< 0.25–28
Picoxystrobin	Fungicide	2	3.0
Piperonyl Butoxide	Synergist	6	1.5–1700
Piraclostrobin	Fungicide	22	0.01–4.6
Pyrethrins	Insecticide	38	< 0.1–14
Pyridaben	Acaricide / Insecticide	2	0.25
Spinosad	Insecticide	2	10
Spirodiclofen	Acaricide (Miticide)	2	1.6
Spiromesifen	Acaricide / Insecticide	10	0.3–1.5

In contrast, 47 of the 50 illegal cannabis samples (94%) tested positive for pesticides, with an average of 3.4 unique pesticides per sample and a total of 24 distinct pesticides identified (Table 3). Myclobutanil was the most prevalent pesticide and was detected in 72% of illegal samples at concentrations up to 130 µg/g. Paclobutrazol was detected in 60% of the samples, whereas chlorfenapyr (24%), fluopyram (24%), boscalid (26%), and pyrethrins (38%) were also commonly detected at significant levels. Piperonyl butoxide was present in only 6% of the samples but reached an exceptionally high concentration of 1700 µg/g in one instance.

### Mycotoxins

Legal cannabis products showed no detectable levels of mycotoxins covered by the method, which covered aflatoxins B1, B2, G1 and G2, as well as ochratoxin A and deoxynivalenol. In contrast, six illegal samples (12%) tested positive for mycotoxins, with three samples containing ochratoxin A and three containing deoxynivalenol (DON, also known as vomitoxin) (Table 4).

**Table 4:** Mycotoxin levels in illegal cannabis samples

Mycotoxin	Concentration (ng/g)
Ochratoxin A	Present*
	18.6 ± 2.6
	23.6 ± 3.3
Deoxynivalenol	Present*
	70.8 ± 8.5
	119 ± 14

* Insufficient quantity available to confirm exact levels in these samples

### Microbial

Microbial contaminant testing in legal cannabis revealed that 20% (*n* = 10) of the samples exceeded the European Pharmacopoeia limits (*Microbiological quality of herbal medicinal products for oral use and extracts used in their preparation*,* general chapter*, 2024) (Table 5). Illegal cannabis samples exhibited significantly greater microbial contamination, frequently surpassing these regulatory thresholds. Specifically, 55% of the illegal samples exceeded the TAMC limit, with counts reaching up to 9 million CFU/g, whereas only 6% of legal products did. Additionally, the TYMC and BGTN limits were exceeded in 73% and 43% of the illegal samples, respectively. Notably, pathogenic contaminants such as *Escherichia coli* were detected exclusively in illegal products, whereas *Aspergillus* spp. occurred at twice the frequency in illegal samples compared with legal samples. Microorganisms found in dried cannabis samples exceeding the European Pharmacopoeia limits were identified via MALDI‒TOF spectrometry (Microflex LT/SH, Bruker Daltonics GmbH & Co. KG, Bremen, Germany) and are listed in Table 6.

**Table 5:** Microbial contaminant levels in legal and illegal dried cannabis

Contaminant	Ph. Eur. Limit (Microbiological quality of herbal medicinal products for oral use and extracts used in their preparation, general chapter, 2024)	Legal cannabis		Illegal cannabis*	
		> Ph. Eur. limit %	Range	> Ph. Eur. limit %	Range
Total aerobic microbial count (TAMC)	100,000 CFU**/g	6	< 100,000 − 300,000 CFU/g	55	< 100,000 − 9,000,000 CFU/g
Total yeast and mold count (TYMC)	10,000 CFU/g	6	< 10,000 - 750,000 CFU/g	73	< 10,000 - 10,000,000 CFU/g
Bile-tolerant gram-negative bacteria (BTGN)	10,000 PNB**/g	6	< 10 - > 10,000 PNB/g	43	< 10 - > 10,000 PNB/g
*E. coli*	Absent	0		2	
*Salmonella* spp.	Absent	0		0	
*Aspergillus* spp.	N/A	14		37	

*n = 49, as only 49 of the 50 illegal samples had sufficient amounts to be tested for microbial contaminants

** abbr: Ph. Eur. =European Pharmacopoeia; CFU = colony forming units; PNB = probable number of bacteria

**Table 6:** Bacteria and *Aspergillus* spp. identified in dried cannabis exceeding Pharmacopoeia limits

Microorganism	Number of occurrence		
	legal cannabis	illegal cannabis	Total
Bacteria			
* Citrobacter amalonaticus/farmeri*	0	1	1
* Citrobacter braakii*	0	1	1
* Citrobacter freundii complex*	0	6	6
* Citrobacter youngae*	0	1	1
* Cronobacter dublinensis*	0	2	2
* Cronobacter sakazakii*	0	2	2
* Dermacoccus nishinomiyaensis*	0	1	1
* Dietzia kunjamensis/maris/schimae*	0	1	1
* Enterobacter cloacae complex*	1	33	34
* Enterobacter hormaechei*	1	2	3
* Enterobacter quasiroggenkampi*	0	1	1
* Enterobacter roggenkampii*	1	3	4
* Enterobacter soli or Enterobacter bugandensis*	0	1	1
* Enterobacterales*	0	1	1
* Enterobacteriaceae Leclercia adecarboxylata*	1	12	13
* Enterococcus durans*	0	1	1
* Enterococcus faecalis*	0	1	1
* Enterobacter tamurae*	0	1	1
* Erwinia aphidicola/persicina*	0	3	3
* Erwinia gerundensis*	0	3	3
* Escherichia albertii/Escherichia coli/Echerichia fergusonii/* * Escherichia marmotea/Shigella boydii/Shigella dysenteriae/* * Shigella flexneri/Shigella sonnei*	0	3	3
* Escherichia hermannii*	0	4	4
* Franconibacter helveticus*	0	3	3
* Franconibacter pulveris*	0	1	1
* Gordonia aichienses/otitidis/sputi*	0	1	1
* Klebsiella (raoultella) aerogenes*	0	1	1
* Klebsiella michiganensis*	0	1	1
* Klebsiella oxytoca/Raoultella ornithinolytica/planticola/terrigena*	0	11	11
* Klebsiella pneumoniae*	0	7	7
* Klebsiella variicola*	0	3	3
* Kosakonia cowanii*	1	8	9
* Mixta calida/gaviniae/intestinalis*	0	7	7
* Pantoea agglomerans*	1	2	3
* Pantoea brenneri*	0	2	2
* Pantoea dispersa*	1	1	2
* Pantoea eucrina*	0	3	3
* Pantoea septica*	1	0	1
* Pantoea vagans*	0	2	2
* Pantoea wallisii*	0	1	1
* Phytobacter ursingii*	0	3	3
* Pseudescherichia vulneris*	0	10	10
* Pseudomonas (stenotrophomonas) beteli/geniculata/* * hibiscicola/lactitubi/maltophilia*	0	1	1
* Pseudomonas aeruginosa/paraeruginosa*	0	5	5
* Pseudomonas coleopterorum*	1	3	4
* Pseudomonas fluorescens group*	0	6	6
* Pseudomonas japonica*	0	2	2
* Pseudomonas koreensis*	0	1	1
* Pseudomonas mendocina*	0	1	1
* Pseudomonas mosselii*	0	1	1
* Pseudomonas oleovorans*	0	1	1
* Pseudomonas oryzihabitans*	0	2	2
* Pseudomonas parafulva*	0	2	2
* Pseudomonas putida group*	0	13	13
* Pseudomonas ryzihabitans*	1	0	1
* Pseudomonas stutzeri*	1	0	1
* Pseudomonas syringae or savastanoi*	0	1	1
* Pseudomonas xanthomarina*	0	1	1
* Rahnella aceris/aquatilis/brichi/variigena/victoriana/woolbedingensis*	0	2	2
* Siccibacter colletis*	0	2	2
* Siccibacter furicensis*	0	3	3
* Staphylococcus epedermidis*	1	0	1
* Stenotrophomonas bentonitica*	0	1	1
*Aspergillus* spp.			
* Aspergillus austroafricanus/creber/cvjetkovicii/fructur/griseoaurantiacus/* * jensenii/protuberus/puulaauensis/sydowii/tabacinus/tennesseensis/* * venenatus/versicolor*	2	5	7
* Aspergillus brasiliensis/niger/tubigensis/vadensis*	1	6	7
* Aspergillus calidoustus/ustus*	2	3	5
* Aspergillus flavus/oryzae/parasiticus*	0	1	1
* Aspergillus fumigatus*	1	1	2
* Aspergillus glaucus group*	1	0	1
* Aspergillus ochraceus/westerdijkiae*	1	11	12
* Aspergillus tamarii*	1	0	1

### THC and contaminant correlation analysis

Across the 100 cannabis samples (50 legal and 50 illegal), detection frequencies varied substantially by analyte class, with metals showing the highest detection rates, while pesticides and microbial indicators exhibited pronounced censoring. To account for non-detects, associations between THC and other analytes were evaluated using a two-part correlation framework that separately assessed co-occurrence (detection vs. non-detection) and concentration relationships conditional on detection. No statistically significant associations between THC and any metal, pesticide, mycotoxin, or microbial analyte were observed after false discovery rate adjustment in either the occurrence or conditional concentration analyses. Several metals, including lithium, cobalt, and nickel, displayed moderate negative rank correlations with THC concentration in detected-only analyses; however, these associations did not remain significant after correction for multiple comparisons. Microbial indicators and pesticides showed weak and inconsistent associations with THC across both analytical components. Stratified analyses by legal status yielded similar patterns, with no reproducible THC-associated correlation structure identified in either legal or illegal samples. This reflects the heterogeneous nature of contaminant occurrence across samples. THC concentration did not exhibit consistent correlations with metals, pesticides, mycotoxins, or microbial indicators in either legal or illegal samples, suggesting that cannabinoid potency was largely independent of contaminant burden in this dataset.

### Cross-contaminant multivariate correlation analysis

**Fig. 4:**
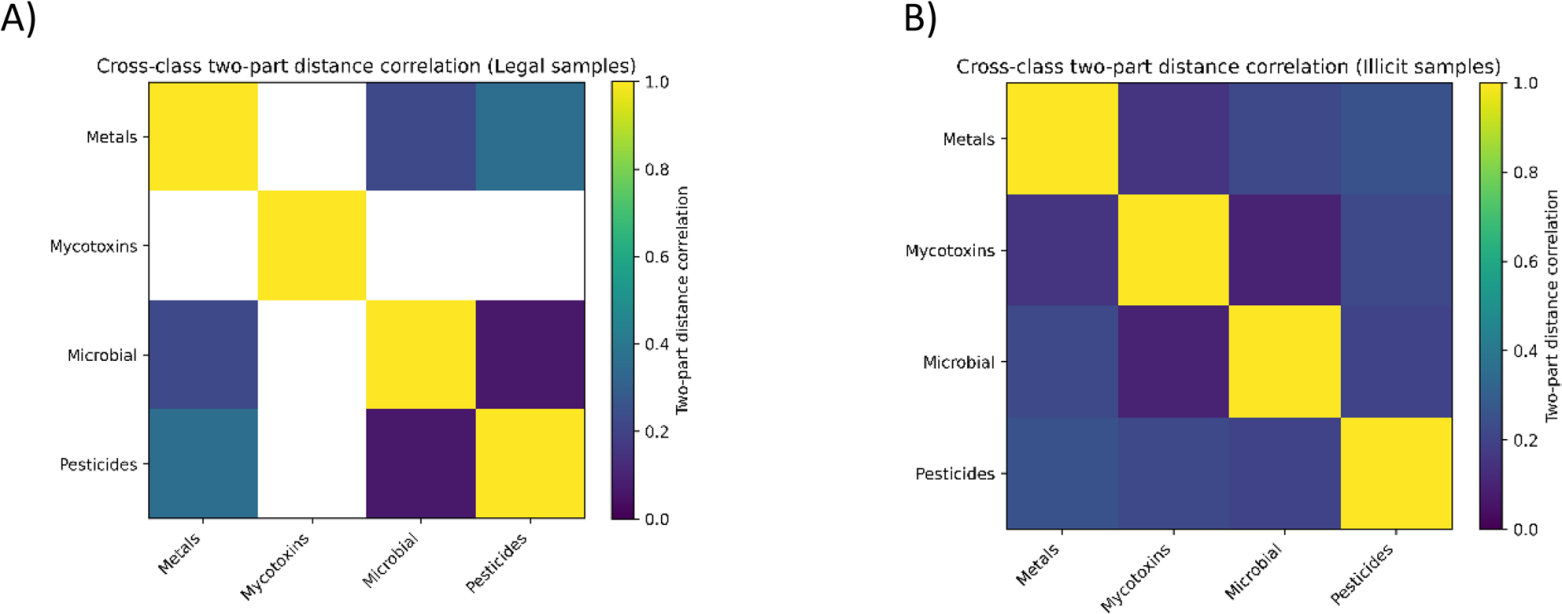
Cross-class associations between contaminant groups in legal (**A**) and illegal (**B**) cannabis samples. Heatmaps show two-part distance correlations integrating detection probability and log-transformed concentration magnitude. Asterisks denote FDR-significant associations (q ≤ 0.05)

Across the 100 samples (50 legal, 50 illegal), non-detects were treated as left-censored values using a two-part framework separating detection frequency from concentration magnitude. Detection-level associations between analytes across contaminant classes (metals, pesticides, mycotoxins, microbial) were evaluated using φ coefficients with Fisher’s exact tests and false discovery rate control. Concentration-level correlations were assessed using Spearman rank correlations on log-transformed detected values, restricted to analyte pairs with sufficient co-detection (*n* ≥ 5). Overall, cross-class correlation signals were limited by sparse co-detection, particularly for mycotoxins and many pesticides, resulting in few analyte pairs suitable for concentration-based inference in the legal group. After FDR correction, detection-level cross-class associations were not statistically significant (Fig. 4). In the concentration layer, one metal–microbial association in the legal group showed a strong positive correlation among co-detected samples (Cu vs. TAMC; q < 0.01), while other observed cross-class correlations in the illegal group were not significant after multiplicity adjustment (Supplementary Table 3).

## Discussion

The primary objective of this study was to broaden the current understanding of cannabis inflorescence THC levels and contamination beyond previously emphasized pesticide residues (Gagnon et al. 2023; Moulins et al. 2018) by incorporating analysis of total THC concentrations, metals, mycotoxins, and microbial contaminants. To achieve this goal, a total of 100 cannabis inflorescence samples, comprising 50 regulated (legal) and 50 unregulated (illegal) products, were collected from Canada. These samples were analyzed via validated, ISO/IEC 17025-accredited/attested methodologies within Health Canada laboratories.

### THC

THC concentrations in legal cannabis inflorescence indicate that approximately 50% of the potency levels measured fall within 20% of their labeled THC content. However, a substantial subset (48%) of legal products demonstrates total THC concentrations deviating by more than 20% below the labeled THC value. This discrepancy has also been reported in other regulatory jurisdictions (Schwabe et al. 2023) (Geweda et al. 2024) (Giordano et al. 2025). Currently, Health Canada lacks a defined regulatory threshold for permissible variability in total THC concentrations in legal inflorescence cannabis products (Cannabis Regulations, 2018). Longitudinal studies have shown that THC can degrade up to 17% after one year of storage (Ross and Elsohly 1997), influenced by factors such as storage duration and exposure to light (Zamengo et al. 2019). Preliminary data from a THC stability study in cannabis inflorescence performed by Health Canada revealed that no significant THC degradation occurred during the first year of storage (data not shown).

Although the exact source of the discrepancies is difficult to determine, it is plausibly multifactorial, involving the absence of standardized sampling and analytical methodologies, limited regulatory enforcement, and commercial incentives favoring elevated THC potency claims. The presence of inaccurate THC labeling adversely affects consumers’ ability to precisely dose cannabis products, undermines perceptions of product quality within the legal market, and may diminish consumer confidence. Accurate THC potency labeling is critical for mitigating risks such as overconsumption, improper dosing, and associated adverse health outcomes. This is particularly important for medical cannabis patients, for whom precise dose titration is essential for therapeutic efficacy and safety.

For samples sourced from the illegal market, the observed variability in THC concentrations may, at least in part, be attributable to differences in sample freshness at the time of seizure. However, it is not feasible to draw definitive conclusions regarding the typical potency of legal versus illegal dried cannabis products, as this study was limited to legal products containing up to approximately 250 mg/g total THC. Furthermore, illegal market samples were submitted without accompanying information regarding their labeled THC content, geographic origin, or date of seizure, precluding direct comparisons. Importantly, the sample set analyzed in this study may not be fully representative of the broader spectrum of legal and illegal cannabis products available on the Canadian market. As such, caution should be exercised in generalizing these findings beyond the specific samples included in this analysis.

### Metals

Cannabis is classified as a hyperaccumulator species characterized by its ability to absorb and concentrate metals from the environment at levels several orders of magnitude greater than those of other plant species (Bengyella et al. 2022; Girdhar et al. 2014). This capacity explains the ubiquitous detection of certain metals, such as copper and molybdenum, in legal and illegal samples. Copper was detected in all cannabis inflorescences, with 100% of legal and 98% of illegal products exceeding the USP concentration limit for inhaled products (3.0 µg/g). Molybdenum was also prevalent and was found in nearly all the samples, with 74% of the legal products exceeding the USP threshold (1.0 µg/g), whereas it was present in 16% of the illegal products. A clear health risk could not be confirmed with the elevated Cu and Mo results (Achuthan et al. 2026). These elevated levels, especially in legal samples, may be attributable to the use of fertilizers and micronutrient additives in commercial cultivation practices. 

Epidemiological studies conducted prior to cannabis legalization reported that individuals who consumed cannabis in the preceding 30 days presented elevated biomarkers of metal exposure compared with nonusers (McGraw et al. 2023). Specifically, compared with nonusers, cannabis users had mean cadmium concentrations that were 22% higher in blood and 18% higher in urine, whereas lead concentrations were 27% higher in blood and 21% higher in urine (McGraw et al. 2023). Given the capacity for metals to be absorbed via inhalation of cannabis smoke and the significant concentrations of arsenic, cadmium, lead, and mercury detected in illegal cannabis inflorescence, consumers of legal cannabis products are likely to experience reduced exposure to these toxic metals. This reduction can be attributed to the to the existing heavy metal testing requirements enforced by Health Canada (Cannabis Regulations, 2018). Both lead and cadmium are toxic at very low concentrations: the U.S. Environmental Protection Agency (EPA) considers any level of lead exposure to pose a health risk, and cadmium is classified as a probable human carcinogen (Genchi et al. 2020). Although arsenic, cobalt, manganese, and mercury were detected at concentrations exceeding USP concentration limits in some cannabis samples, previous studies have demonstrated no statistically significant associations between cannabis use and elevated levels of these metals in biological matrices (McGraw et al. 2023). This suggests a relatively low systemic absorption rate of these elements via smoke inhalation. A risk assessment case study on the metal results of the 50 legal cannabis products has been recently published by Achuthan et al. 2026.

### Pesticides

Pesticide residue analysis of legal and illegal cannabis inflorescence corroborates the findings of (Gagnon et al. 2023), and the expanded sample size in the present analysis provides a more robust national representation of pesticide usage patterns across both the legal and illegal markets. The overall pesticide detection rate was 4% in legal samples and 94% in illegal samples, which is consistent with prior results. For legal products, the same two pesticides—dichlobenil and myclobutanil—were detected at the LCL of 0.01 µg/g. Such levels in legal products are likely due to environmental or accidental contamination rather than intentional use. For the legal samples, appropriate compliance and enforcement measures were taken to address each of these cases. Although dichlobenil was detected and quantified in a single legal cannabis sample, it is not included in the mandatory pesticide active ingredient panel specified by the *Mandatory Cannabis Testing for Pesticide Active Ingredients Requirements* (Mandatory cannabis testing for pesticide active ingredients requirements, 2019). This gap highlights the importance of expanded multiresidue analytical methods with enhanced sensitivity and selectivity to capture a broader spectrum of pesticide residues.

In contrast, illegal cannabis samples yielded both previously identified and newly quantified pesticide residues. Notably, the newly detected compounds included p, p’-dicofol, dimethoate, fluxapyroxad, metalaxyl, n-octyl bicycloheptene dicarboximide, oxathiapiprolin, picoxystrobin, and piraclostrobin, with the latter showing a substantial sample positivity rate of 22%.

Consistent with earlier findings (Gagnon et al. 2023), myclobutanil, paclobutrazol, and pyrethrins were detected at high frequencies, ranging from 38% to 72% across illegal samples. Furthermore, notable shifts in the prevalence of specific pesticides were observed. The sample positivity rates for boscalid, chlorfenapyr, and fluopyram increased by approximately 6- to 12-fold compared with previous data, whereas the detection frequency of piperonyl butoxide decreased approximately threefold. The maximum concentrations of several pesticides—including boscalid, fluopyram, myclobutanil, permethrin, piperonyl butoxide, pyrethrins, and spinosad—in illegal samples were found to be 1 to 3 orders of magnitude higher than those reported in (Gagnon et al. 2023). The high levels and type of pesticides detected suggest that these were highly likely to have been intentionally applied during illegal cultivation.

### Microbial contaminants and mycotoxins

Cannabis plants interact with a community of microbes consisting of more than 100 species of fungi and bacteria that can produce toxic secondary metabolites (e.g., mycotoxins) that can potentially affect the health of humans (Gwinn et al. 2023).

Testing for microbial contaminants revealed that 20% of legal samples and a significantly greater proportion (43–55%) of illegal samples contained microbial levels above the European Pharmacopoeia limits. Production sites and agroclimatic conditions have previously been shown to significantly influence microbial load and composition (Sopovski et al. 2023), which could explain the higher bacterial and fungal prevalence in illegal samples not produced in controlled environments and not subjected to the stringent quality standards of the legal market (Cannabis Regulations, 2018). The most commonly identified genera of bacteria included species of *Enterobacter*,* Klebsiella*,* Pseudomonas* and *Pantoea*, with notable differences in their presence between legal and illegal samples, with the former being more common in illegal samples (Table 6). *Enterobacter cloacae* was detected in 72% of illegal samples and is recognized in the literature as an opportunistic pathogen (Hennigs et al. 2011). *Klebsiella* spp. and *Pseudomonas aeruginosa*, which are associated with respiratory infections (Bahy et al. 2022), were also identified predominantly in illegal samples. For the 20% of regulated samples that exceeded the limit, appropriate compliance and enforcement measures were taken to address each of these cases, which resulted in voluntary recall in one instance.

Similarly, the significantly higher TYMC and *Aspergillus* counts in illegal samples than in legal products may increase health risks, as inhalation of their spores can cause lung infections, particularly in immunocompromised and cancer patients, in whom aspergillosis accounts for 43% of fungal infections associated with cannabis use (Benedict et al. 2020). Species of *Aspergillus* are known to produce ochratoxin A, which can result in kidney damage (Gwinn et al. 2023). The observed prevalence of ochratoxin A in illegal cannabis inflorescence samples corroborates previous findings in California (Jameson et al. 2022) and Luxembourg (Buchicchio et al. 2022). Ochratoxin A in illegal cannabis can be of concern, as although heat combustion can degrade a portion of ochratoxin A, it does not always result in reduced toxicity since some degradation products are as toxic as the parent molecule (Boudra et al. 1995). Similarly, the *Fusarium* mycotoxin DON has been shown to accumulate in cannabis inflorescence and to cause chronic immunosuppression, cancer and toxicosis (Gwinn et al. 2023). Even though the smoking process can reduce the level of DON, this mycotoxin is efficiently and rapidly absorbed into the bloodstream through inhalation. However, there are no tolerance limits established in any of the publications listed in Schedule B of the *Food and Drugs Act* for these two contaminants in cannabis (Sarma et al. 2020).

### Multivariate correlation analysis

Multivariate correlation results indicate that co-occurrence of contaminants in cannabis products is sporadic rather than systematic, and that contamination profiles are largely independent across chemical and biological classes. These findings support the interpretation that observed contaminant presence likely reflects distinct sources and mechanisms rather than shared pathways linked to THC concentration or a single contamination process.

## Conclusion

This study is a comprehensive assessment of contaminant profiles in both legal and illegal cannabis inflorescences in Canada, encompassing not only pesticide residues (Gagnon et al. 2023; Moulins et al. 2018) but also metals, mycotoxins, microbial contaminants, and THC concentration variability. Notably, 48% of legal cannabis products presented THC concentrations deviating by more than 20% from their labeled values. Such labeling inaccuracies can compromise dosing precision, undermine consumer confidence in regulated products, and pose significant safety concerns, particularly for medical cannabis users.

Microbiological analysis revealed that 20% of legal samples exceeded the European Pharmacopoeia microbial limits, prompting appropriate compliance and enforcement measures to address each of these cases. Illegal samples presented substantially elevated contamination, with 55% surpassing aerobic microbial limits and 73% exceeding yeast and mold thresholds. Mycotoxins were undetected in legal products but were present in 12% of illegal samples.

Pesticide analysis identified trace residues of myclobutanil and dichlobenil at the lowest calibrated level (0.01 µg/g) in only two legal samples, whereas 94% of illegal samples tested positive for pesticides, averaging 3.4 unique residues per sample, encompassing 24 distinct compounds.

Metal quantification demonstrated a significantly greater prevalence and concentrations of arsenic, cadmium, lead, and mercury in illegal cannabis than in legal products. The legal samples, however, presented greater chromium levels, with peak concentrations nearly threefold higher than those of illegal cannabis. The concentrations of arsenic, barium, chromium, copper, molybdenum, nickel, and vanadium exceeded the USP tolerance limits in one or both groups.

Collectively, these findings provide critical data to inform regulatory policy enhancements and support evidence-based decision-making among Canadian cannabis consumers.

## Supplementary Information

Supplementary Material 1.

Supplementary Material 2.

## Data Availability

The data is available from the corresponding author on request.

## Abbreviations

LC-MS/MS: Liquid chromatography – triple quadruple mass spectrometry
UV: Ultraviolet
MS: Mass spectrometry
THC: Tetrahydrocannabinol
TAMC: Total aerobic microbial count
TYMC: Total yeast and mold count
BTGN: Bile-tolerant gram-negative bacteria
USP: United States Pharmacopeia
LCL: Lowest calibrated level
Ph Eur: European Pharmacopoeia
CFU: Colony-forming units
PNB: Probable number of bacteria

## Footnotes

1 The methodologies presented herein are intended exclusively to advance scientific understanding. Utilization of these methods outside the context of a well-established and robust quality management system, encompassing rigorous controls, verification procedures, and deviation management, may compromise the precision and reproducibility of the results. Consequently, the authors and their affiliated employers disclose any liability arising from the application of these methods.
